# Liraglutide *via* Activation of AMP-Activated Protein Kinase-Hypoxia Inducible Factor-1α-Heme Oxygenase-1 Signaling Promotes Wound Healing by Preventing Endothelial Dysfunction in Diabetic Mice

**DOI:** 10.3389/fphys.2021.660263

**Published:** 2021-08-16

**Authors:** Huiya Huang, Linlin Wang, Fanyu Qian, Xiong Chen, Haiping Zhu, Mei Yang, Chunxiang Zhang, Maoping Chu, Xiaorong Wang, Xiaozhong Huang

**Affiliations:** ^1^The First Affiliated Hospital of Wenzhou Medical University, Wenzhou, China; ^2^The Second Affiliated Hospital and Yuying Children’s Hospital of Wenzhou Medical University, Wenzhou, China

**Keywords:** Liraglutide, diabetic wound healing, endothelial dysfunction, Heme oxygenase-1, 5'-AMP activated kinase, hypoxia inducible factor-1α

## Abstract

**Background/Aims**: Diabetic foot ulcers (DFUs) present a major challenge in clinical practice, and hyperglycemia-induced angiogenesis disturbance and endothelial dysfunction likely exacerbate DFUs. The long-acting glucagon-like peptide-1 (GLP-1) analog liraglutide (Lira) is a potential activator of AMP-activated protein kinase (AMPK) that appears to enhance endothelial function and have substantial pro-angiogenesis and antioxidant stress effects. Therefore, in this study, we aimed to investigate whether the protective role of Lira in diabetic wound healing acts against the mechanisms underlying hyperglycemia-induced endothelial dysfunction and angiogenesis disturbance.

**Methods**: Accordingly, db/db mice were assessed after receiving subcutaneous Lira injections. We also cultured human umbilical vein endothelial cells (HUVECs) in either normal or high glucose (5.5 or 33 mM glucose, respectively) medium with or without Lira for 72 h.

**Results:** An obvious inhibition of hyperglycemia-triggered endothelial dysfunction and angiogenesis disturbance was observed; follow by a promotion of diabetic wound healing under Lira treatment combined with restored hyperglycemia-impaired AMPK signaling pathway activity. AMPKα1/2 siRNA and Compound C (Cpd C), an inhibitor of AMPK, abolished both Lira-mediated endothelial protection and pro-angiogenesis action, as well as the diabetic wound healing promoted by Lira. Furthermore, hypoxia inducible factor-1α (Hif-1α; transcription factors of AMPK substrates) knockdown in HUVECs and db/db mice demonstrated that Lira activated AMPK to prevent hyperglycemia-triggered endothelial dysfunction and angiogenesis disturbance, with a subsequent promotion of diabetic wound healing that was Hif-1α–heme oxygenase-1 (HO-1) axis-dependent. Taken together, these findings reveal that the promotion of diabetic wound healing by Lira occurs *via* its AMPK-dependent endothelial protection and pro-angiogenic effects, which are regulated by the Hif-1α–HO-1 axis.

## Introduction

In the past 3 decades, the number of people diagnosed with diabetes mellitus globally has quadrupled, with approximately one in 11 adults now having this condition (Zheng et al., 2018). Among the serious complications caused by diabetes mellitus, diabetic foot ulcers (DFUs), which are caused by decreased wound healing in the lower extremities, can be some of the most severe and difficult to treat. Despite their high healthcare costs, current treatments for DFUs have significant side-effects and often result in non-compliance. Approximately 20% of diabetes patients with DFUs do not heal within 1 year, even after wound resolution; moreover, with a recurrence rate of roughly 40% within a year, subsequent DFUs are quite common (Prompers et al., 2008; Armstrong et al., 2017; McLaughlin et al., 2017). Thus, more effective therapeutic methods are urgently needed.

Impaired angiogenesis is a critical factor that impedes the healing of DFUs (Ackermann et al., 2014). Given that blood vessels supply tissues with oxygen and nutrients, angiogenesis plays a crucial role in wound healing (Chen et al., 2018). Thus, the improvement of angiogenesis is a key aim of DFU therapy. The endothelial cells that line blood vessels are crucial to angiogenesis and consequently the wound healing process (Sawada et al., 2014). However, one of the earliest and most prominent changes associated with the onset of diabetes is endothelial dysfunction, which is also recognized as an initial event in the progression of many serious diabetic complications (Wils et al., 2017).

Prolonged increases in reactive oxygen species (ROS) in the vasculature are a major contributor to diabetic endothelial dysfunction (Tousoulis et al., 2013). Liraglutide (Lira) has been shown to be an effective antioxidant (Wang et al., 2016a), and it has attracted increasing scientific attention owing to its cardiovascular benefits. For example, Lira has been demonstrated to both modulate endothelial function and prevent hyperglycemia-induced vascular abnormalities, and this effect appears to be independent of its effectiveness in controlling blood glucose levels (Cariou, 2012). However, there has been little published research on the effect of Lira on DFUs and patient outcomes, let alone the mechanisms mediating these effects.

The conserved energy sensor AMP-activated protein kinase (AMPK) is expressed in endothelial cells of various origins, and emerging evidence has implicated AMPK in regulating vascular homeostasis (Omura et al., 2016). Activation of AMPK in endothelial cells can trigger several biological effects that promote vascular homeostasis, including suppression of hyperglycemia-induced generation of ROS, alleviation of free fatty acid–induced lipotoxicity, and protection against apoptosis (Li et al., 2012). Furthermore, many studies have demonstrated a role for AMPK activation in angiogenesis and diabetic wound healing (Lin et al., 2014; Abdel Malik et al., 2017).

In at least several cell types, Lira has been observed to activate AMPK (Inoue et al., 2015; Jojima et al., 2017). The present study aimed to determine whether Lira enhances diabetic wound healing through its AMPK-dependent anti-ROS and pro-angiogenic effects. We demonstrate that AMPK activity is crucial to the beneficial effect of Lira on endothelial function and diabetic wound healing through promoting HIF-1α transcriptional activity and expression of its target hemoxygenase-1 (HO-1), thus elucidating an additional mechanism by which Lira mediates diabetic wound healing.

## Materials and Methods

### Cell Culture

Human umbilical vein endothelial cells (HUVECs) obtained from Lonza were maintained and cultured as previously described by Sun et al. (2021). Briefly, HUVECs were cultured in normal glucose (NG, 5.5 mM of glucose), high glucose (HG, 33 mM of glucose), or osmotic control conditions (27.5 mM of d-mannitol and 5.5 mM of glucose) with or without Lira (100 nM; Selleck, S8256) for 72 h, with the media changed every 24 h. Cells were pretreated with 10 μM of Compound C (Cpd C; Selleck, S7840) and 5 μM of 2-methoxyestradiol (2-ME; Selleck, S1233), inhibitors of AMPK and Hif-1α, respectively, for 2 h every day before Lira administration for pathway signal analysis. After being dissolved in DMSO, inhibitors were diluted to their final concentration with sterile saline (final DMSO concentration, <2‰). *n* = 5 for each experiment.

### Animal Procedures

Diabetic db/db mice as well as their control db/m littermates were obtained from Jackson Laboratories (Strain: BKS.Cg-Dock7^m+/+^Lepr^db/J^), and C57BL/6 mice were procured from the Model Animal Research Center of Nanjing University. All mice were maintained under a 12-h light/dark cycle at 22 ± 2°C and 60 ± 5% relative humidity. All animal experiments and procedures in this study followed ethical guidelines for animal studies and were approved by the Institutional Animal Care and Use Committee of Wenzhou Medical University, China.

For long-term systemic treatment, Lira (Selleck, S8256) was administered to 8-week-old mice at a dose of 200 μg/kg/day for 4 weeks (Fujita et al., 2014), and saline served as the vehicle control. Analyses were performed following a 4-week course of treatment. At the end of treatment period, 4-h fasting blood glucose levels from the tail vein were determined (Supplementary Figure S1). For the signaling pathway analysis, pathway antagonist: Cpd C (Selleck, S7840), an inhibitor of AMPK, was administered at the dose of 1.5 mg/kg/day (Saldivia et al., 2016). 2-ME, an inhibitor of Hif-1α (Selleck, S1233), was administered at the dose of 40 mg/kg/day (Gerby et al., 2016). All these reagents were administered to the mice using subcutaneously embedded osmotic minipumps (ALZET, 1002; DURECT, Cupertino, CA). *n* = 5 for each experiment.

### *In vivo* Wound Model

An *in vivo* wound model was utilized as described by Sun et al. (2021) following the same procedure to create the wound model and wound healing assay. Briefly, general anesthesia was performed with 2% inhaled isoflurane and then injected subcutaneously with the analgesic. The hair of the back was shaved with an electric clipper followed by a depilatory cream. The skin was rinsed with alcohol, and two full-thickness wounds were made using a 6-mm biopsy punch on the dorsum on each side of the midline. Of the two wounds created, one was smeared with Lira (100 nM), and the other was treated with 50 μl of saline as an internal control. The 4-h fasting blood glucose levels from the tail vein were determined (Supplementary Figure S1). For the signaling pathway analysis, the wounds smeared with Lira received either 10 μM of Cpd C or 5 μM of 2-ME, injected at the edges of wounds immediately and 4 days after wounding (Li et al., 2015). Each wound was photographed at the indicated time. Animals were sacrificed 7 or 14 days post-wounding, and wounds were harvested and fixed in 10% neutral buffered formalin for the CD31 immunofluorescence analyses. *n* = 5 for each experiment.

### Statistical Analysis

Results are presented as mean ± SEM values. For comparisons of two experimental groups, statistical differences between treatment means were assessed using unpaired two-tailed Student’s *t*-tests, whereas differences in treatment means among three or more groups were assessed using one-way ANOVA. Bonferroni’s *post hoc* testing was employed after ANOVA for testing for significant differences between groups. A two-tailed *p* value of less than 0.05 was considered statistically significant. Statistical analyses were done using GraphPad Prism (GraphPad Software).

## Results

### Lira Attenuates Hyperglycemia-Induced Endothelial Dysfunction Both *in vivo* and *in vitro*

**Figure 1:**
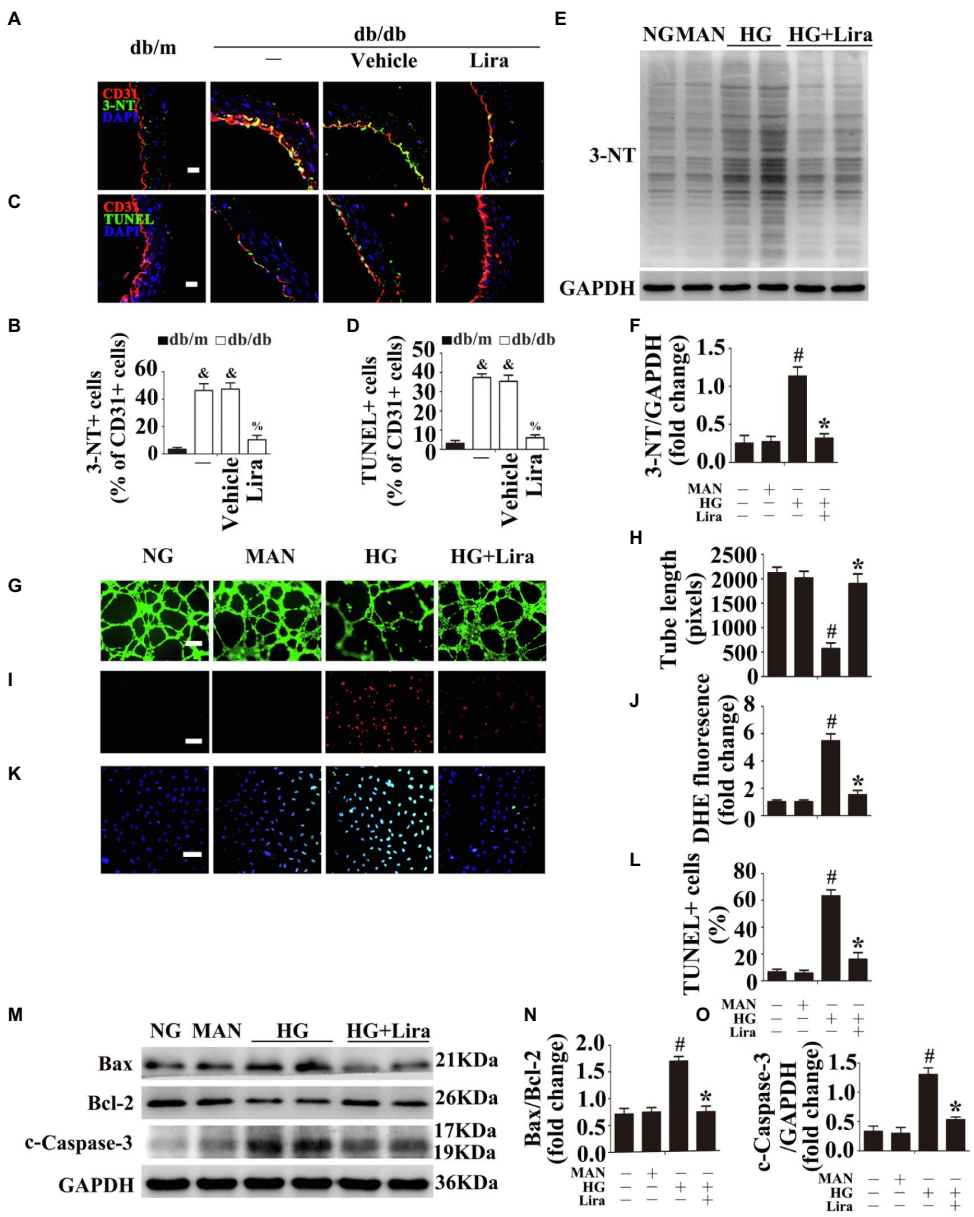
Liraglutide (Lira) attenuates hyperglycemia-induced endothelial dysfunction both *in vivo* and *in vitro*. **(A)** The representative images of immunofluorescence with 3-NT, scale bars = 20 μm and **(**C**)** endothelial cells TUNEL assay, scale bars = 20 μm, from db/m mice, db/db mice, and db/db mice receiving Lira (200 μg/kg/day) or vehicle treatment with saline infusion aorta tissue sections. **(B)** Quantification of the proportions of 3-NT positive cells and **(D)** TUNEL-positive cells of CD31+ cells. **(E)** Cell lysates of human umbilical vein endothelial cells (HUVECs) were used to detect the 3-NT protein levels by immunoblotting. HUVECs cultured in different mediums containing normal glucose (NG; 5.5 mM), high glucose (HG; 33 mM) alone or with Lira (100 nM) for 72 h, and mannitol (MAN; 33 mM: 5.5 mM of glucose + 27.5 mM of D-mannitol) served as the osmotic control for the HG. **(F)** The quantitative analysis of the immunoblots. **(G)** Capillary-like tube formation, scale bars = 300 mm. **(I)** Fluorescence with DHE, scale bars = 100 mm and **(K)** TUNEL assay, scale bars = 100 mm, HUVECs were treated as indicated in **(E)**. **(H)** Quantification of the tube length, **(J)** the DHE fluorescence intensity ratio, and **(L)** the quantitative analysis of TUNEL cells. **(M)** Cell lysates of HUVECs were used to detect the Bax, Bcl-2, and c-Caspase-3 protein levels by immunoblotting. HUVECs treated as indicated in **(E)**. **(N,O)** The quantitative analysis of each immunoblot. All values displayed are means ± SEM of five independent experiments. ^&^*p* < 0.05 vs. db/m mice; ^%^*p* < 0.05 vs. db/db mice or vehicle treated db/db mice. ^#^*p* < 0.05 vs. NG or MAN; and ^*^p < 0.05 vs. HG.

Prolonged increases in ROS in the vasculature are a major contributor to diabetic endothelial dysfunction (Wang et al., 2016a). Thus, immunofluorescence staining for the oxidative damage marker 3-NT revealed high levels of oxidative stress in the aortic endothelium of diabetic db/db mice compared with their corresponding db/m control littermates. Systemic treatment with Lira significantly attenuated hyperglycemia-induced oxidative stress relative to the vehicle-treated diabetic db/db mice (Figures 1A,B). Moreover, TUNEL staining revealed high levels of apoptosis in the aortic endothelium of db/db mice, with Lira treatment ameliorating hyperglycemia-induced endothelial apoptosis (Figures 1C,D).

Meanwhile, pro-angiogenesis activity was also considerably impaired in HUVECs exposed to HG, as demonstrated by their impaired tube-forming activity (Figures 1G,H), but was largely restored by Lira co-treatment. Furthermore, culturing HUVECs under HG conditions caused a substantial increase in ROS formation in endothelial cells, as demonstrated by increased fluorescence-labeled Dihydroethidium (DHE) staining (Figures 1I,J) and elevated 3-NT protein levels (Figures 1E,F), whereas these effects were largely attenuated by Lira co-treatment. Additionally, HG conditions induced high levels of apoptosis in HUVECs, as demonstrated by increases in TdT‐mediated dUTP nick end labeling (TUNEL)-positive cells (Figures 1K,L), the Bax/Bcl-2 ratio (Figures 1M,N), and c-Caspase-3 protein levels (Figures 1M,O). However, HG-induced apoptosis was significantly alleviated by Lira. Thus, Lira was confirmed to have protective effects against HG-induced endothelial impairment.

### The Endothelial Protective Action of Lira Against Hyperglycemia is AMPK-Dependent

**Figure 2:**
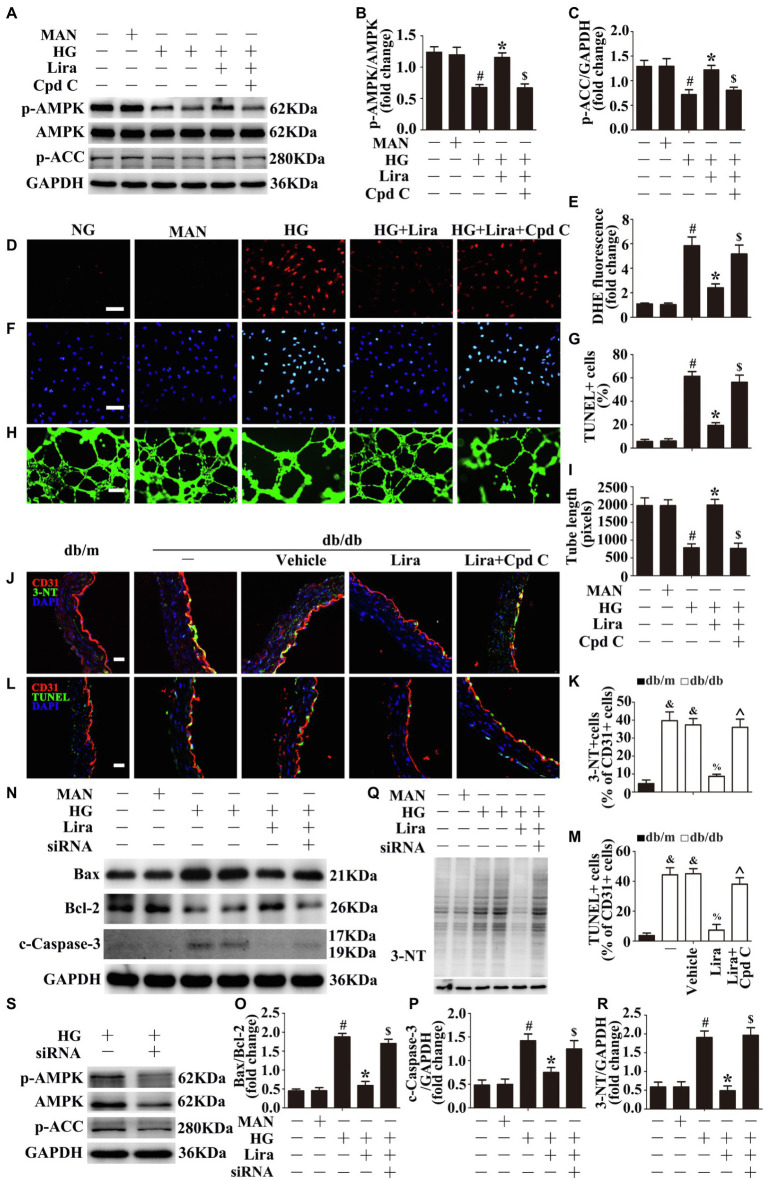
The endothelial protective action of Lira against hyperglycemia is AMP-activated protein kinase (AMPK)-dependent. **(A)** Cell lysates of HUVECs were used to detect the p-AMPK and phosphorylated acetyl-CoA carboxylase (p-ACC) protein levels by immunoblotting. HUVECs were cultured either in NG or HG medium alone or with Lira (100 nM) for 72 h, MAN served as the osmotic control for the HG. For signaling pathway analysis, Compound C (Cpd C) inhibitor of AMPK (10 μM) was pretreated for 2 h before Lira administration. **(B,C)** The quantitative analysis of each immunoblot. **(D)** Fluorescence with DHE, scale bars = 100 mm, **(F)** TUNEL assay, scale bars = 100 mm, and **(H)** capillary-like tube formation, scale bars = 300 mm. HUVECs were treated as indicated in **(A)**. **(E)** Quantification of the DHE fluorescence intensity ratio, **(G)** the quantitative analysis of TUNEL+ cells, and **(I)** the tube length. **(J)** The representative images of immunofluorescence with 3-NT, scale bars = 20 μm and (**L**) endothelial cells TUNEL assay, scale bars = 20 μm, from db/m mice, db/db mice, and db/db mice receiving Lira (200 μg/kg/day) or vehicle treatment with saline infusion aorta tissue sections. For signaling pathway analysis, Cpd C inhibitor of AMPK, was administered at the dose of 1.5 mg/kg/day. **(K)** Quantification of the proportion of 3-NT positive cells and **(M)** TUNEL-positive cells of CD31+ cells. **(N,Q,S)** Cell lysates of HUVECs were used to detect the Bax, Bcl-2, c-Caspase-3, 3-NT, p-AMPK, and p-ACC protein levels by immunoblotting. HUVECs were transfected with si-AMPKα1/α2 or control siRNA, respectively. After transduction, HUVECs were cultured either in NG, or HG medium alone or with Lira (100 nM) for 72 h. **(O,P,R)** The quantitative analysis of each immunoblot. All values displayed are means ± SEM of five independent experiments. ^&^*p* < 0.05 vs. db/m mice; ^%^*p* < 0.05 vs. db/db mice or vehicle-treated db/db mice; ^*p* < 0.05 vs. db/db mice receiving Lira; ^#^*p* < 0.05 vs. NG or MAN; ^*^p < 0.05 vs. HG; ^$^*p* < 0.05 vs. HG co-incubated with Lira.

Emerging evidence implicates AMPK in the regulation of vascular homeostasis and repair processes among individuals with diabetes (Li et al., 2012; Omura et al., 2016). A previous study demonstrated that phosphorylation of AMPK at Thr172 is correlated with activity of AMPK (Rabinovitch et al., 2017; Wang et al., 2016b), thereby affecting Serine79-phosphorylated acetyl-CoA carboxylase (p-ACC^ser79^), a direct downstream target of AMPK. To determine whether Lira activates AMPK, endothelial cells were cultured in either NG or HG conditions with or without Lira for 72 h. We observed impaired AMPK activity in HUVECs exposed to HG, with phosphorylation of AMPK^Thr172^ and ACC^ser79^ decreased. Importantly, Lira largely increased HG-downregulated AMPK activity (Figures 2A–C). Furthermore, pretreatment with Cpd C, a specific AMPK inhibitor, abrogated Lira-mediated AMPK activation effects on HUVECs under HG conditions.

Next, we sought to determine whether the endothelial protective effect of Lira is AMPK-dependent. Pretreatment with Cpd C abrogated Lira-mediated anti-oxidant stress (Figures 2D,E), anti-apoptosis (Figures 2F,G), and pro-angiogenesis (Figures 2H,I) effects in HUVECs under HG conditions. To assess the key role of AMPK in Lira-mediated endothelial protection, siRNA transfection was used to interfere with AMPKα1/2 expression in HUVECs by (Figure 2S). Thus, the previously demonstrated endothelial protective effects of Lira were also abolished, consistent with the observed increases in levels of apoptosis (Figures 2N–P) and oxidative stress (Figures 2Q,R). Moreover, the aortic endothelium samples from db/db mice also exhibited a decrease in Lira-exerted anti-oxidant stress (Figures 2J,K) and anti-apoptosis (Figures 2L,M) effects under Cpd C treatment.

### Downstream of AMPK, Hif-1α Is Involved in the Endothelial Protective Effect of Lira Against Hyperglycemia

**Figure 3:**
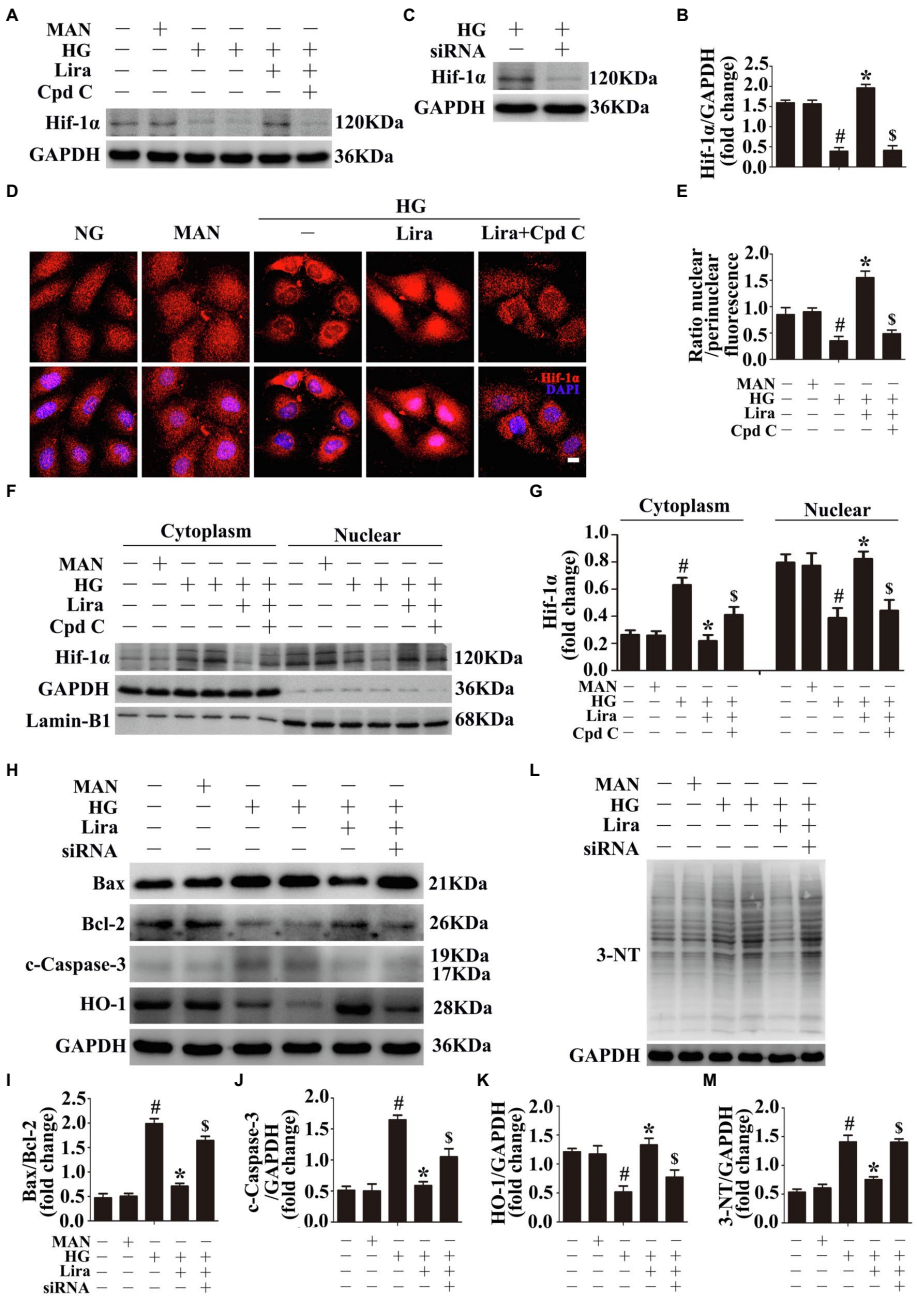
Hypoxia inducible factor-1α (Hif-1α), as the downstream of AMPK, participates in the endothelial protective action of Lira against hyperglycemia, *in vitro*. **(A)** Cell lysates of HUVECs were used to detect the Hif-1α protein levels by immunoblotting. HUVECs were cultured either in NG, or HG medium alone or with Lira (100 nM) for 72 h, MAN served as the osmotic control for the HG. For signaling pathway analysis, Cpd C inhibitor of AMPK (10 μM) was pretreated for 2 h before Lira administration. **(B)** The quantitative analysis of each immunoblot. **(C)** Cell lysates of HUVECs were used to detect the Hif-1α protein levels by immunoblotting. HUVECs were transfected with si-Hif-1α or control siRNA, respectively. After transduction, HUVECs were cultured in HG medium alone for 72 h. **(D)** Representative immunofluorescence with Hif-1α in HUVECs, which treated as indicated in **(A)**. Scale bars = 5 mm. **(E)** The quantitative analysis of nuclear/perinuclear Hif1α fluorescence intensity ratio in **(D)**. **(F)** Nuclear and cytosolic extracts were isolated to detect the Hif-1α protein levels by immunoblotting. HUVECs treated as indicated in **(A)**. **(G)** The quantitative analysis of each immunoblot. **(H,L)** Cell lysates of HUVECs were used to detect the Bax, Bcl-2, c-Caspase-3, and HO-1 protein levels by immunoblotting. HUVECs were transfected with si-Hif-1α or control siRNA, respectively. After transduction, HUVECs were cultured either in NG, or HG medium alone or with Lira (100 nM) for 72 h. **(I,J,K,M)** The quantitative analysis of each immunoblot. All values displayed are means ± SEM of five independent experiments. ^#^*p* < 0.05 vs. NG or MAN; ^*^p < 0.05 vs. HG; ^$^*p* < 0.05 vs. HG co-incubated with Lira.

A previous study has identified transcription factor Hif-1α as an important element downstream of AMPK that mediates the pro-angiogenic effects of AMPK and controls cellular ROS levels (Abdel Malik et al., 2017; Rabinovitch et al., 2017). Recent studies have found that the Hif-1a–HO-1 axis not only has an antioxidant function but is also related to angiogenesis (Choi et al., 2017; He et al., 2018). In HG-treated HUVECs, we observed decreased Hif-1α protein levels relative to NG-treated HUVECs. Lira co-treatment increased Hif-1α protein levels. To demonstrate that Lira-modulated Hif-1α upregulation was attributable to AMPK, HUVECs were treated with the AMPK inhibitor Cpd C. In AMPK-inhibited HUVECs, Lira no longer upregulated Hif-1α expression (Figures 3A,B). We then assessed the subcellular localization of Hif-1α. The cellular nucleic and cytoplasmic fractions of HUVECs were isolated and subjected to immunoblotting. Among HG-treated HUVECs, Hif-1α was localized mainly in the cytoplasm, whereas co-incubation with Lira shifted the distribution into the nucleus. However, Cpd C treatment abolished the Lira-mediated nuclear distribution of Hif-1α in HUVECs (Figures 3F,G). Immunofluorescence staining also confirmed the subcellular localization of Hif-1α (Figures 3D,E).

To assess the key role of the Hif-1α–HO-1 axis in Lira-mediated endothelial protection, siRNA transfection was used to silence Hif-1α expression in HUVECs (Figure 3C). In HG-treated HUVECs, we observed decreased HO-1 protein levels relative to that of NG-treated HUVECs. Lira co-treatment increased the HO-1 protein level; however, in Hif-1α siRNA-transfected HUVECs, Lira no longer upregulated HO-1 expression (Figures 3H,K). In addition, the previously demonstrated endothelial protective effect of Lira was also abolished, consistent with increases in the level of apoptosis (Figures 3H–J) and oxidative stress (Figures 3L,M).

**Figure 4:**
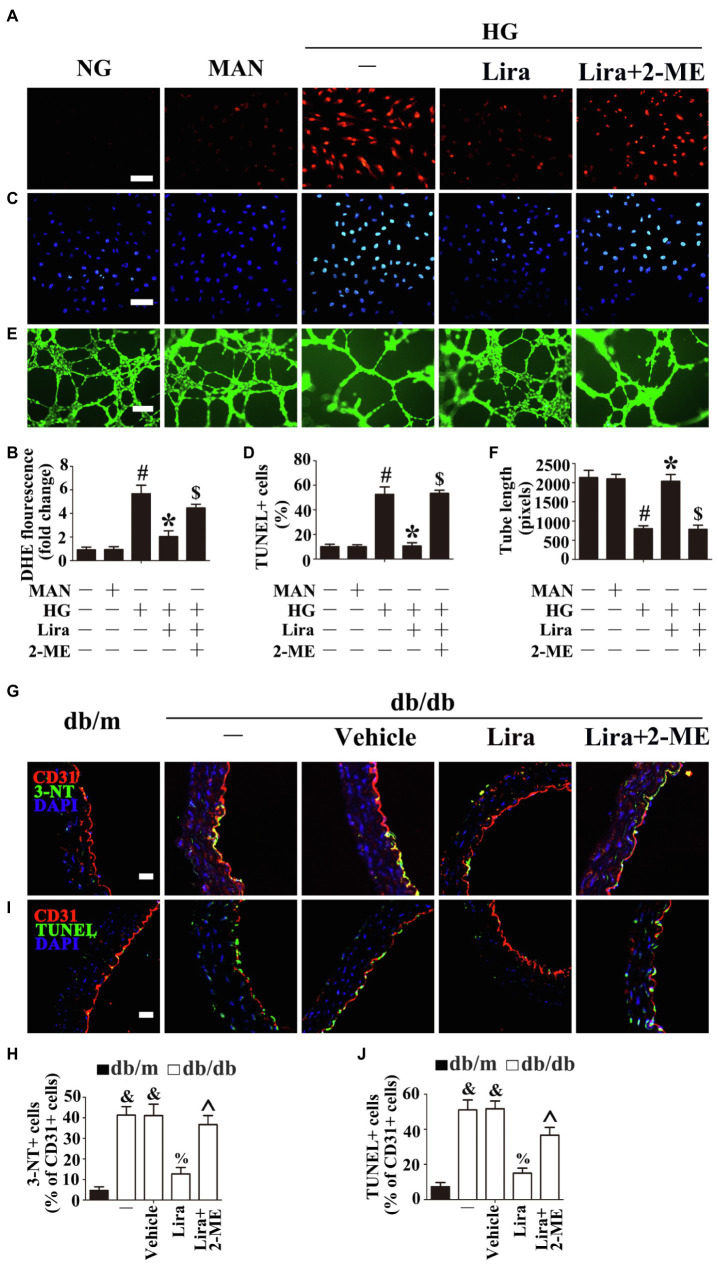
Hif-1α, as the downstream of AMPK, participates in the endothelial protective action of Lira against hyperglycemia, *in vitro* and *in vivo*. **(A)** Fluorescence with DHE, scale bars = 100 mm, **(C)** TUNEL assay, scale bars = 100 mm, and **(E)** capillary-like tube formation, scale bars = 300 mm, HUVECs were cultured either in NG, or HG medium alone or with Lira (100 nM) for 72 h, MAN served as the osmotic control for the HG. For signaling pathway analysis, 2-ME, an inhibitor of Hif-1α (5 μM), was pretreated for 2 h before Lira administration. **(B)** Quantification of the DHE fluorescence intensity ratio, **(D)** the quantitative analysis of TUNEL+ cells, and **(F)** the tube length. **(G)** The representative images of immunofluorescence with 3-NT, scale bars = 20 μm and **(I)** endothelial cells TUNEL assay, scale bars = 20 μm, from db/m mice, db/db mice, and db/db mice receiving Lira (200 μg/kg/day) or vehicle treatment with saline infusion aorta tissue sections. For signaling pathway analysis, 2-ME, an inhibitor of Hif-1α, was administered at the dose of 40 mg/kg/day. **(H)** Quantification of the proportion of 3-NT-positive cells and **(J)** TUNEL-positive cells of CD31+ cells. All values displayed are means ± SEM of five independent experiments. ^&^*p* < 0.05 vs. db/m mice; ^%^*p* < 0.05 vs. db/db mice or vehicle-treated db/db mice; ^^^*p* < 0.05 vs. db/db mice receiving Lira. ^#^*p* < 0.05 vs. NG or MAN; ^*^p < 0.05 vs. HG; ^$^*p* < 0.05 vs. HG co-incubated with Lira.

To confirm this role of the Hif-1α–HO-1 axis in Lira-mediated endothelial protection, HUVECs were treated with 2-ME, a specific Hif-1α inhibitor. Pretreatment with 2-ME abrogated Lira-mediated anti-oxidant stress (Figures 4A,B), anti-apoptosis (Figures 4C,D), and pro-angiogenesis (Figures 4E,F) effects in HUVECs under HG treatment. Meanwhile, the aortic endothelium of db/db mice exhibited a decrease in Lira-exerted anti-oxidant stress (Figures 4G,H) and anti-apoptosis (Figures 4I,J) effects under the 2-ME treatment.

### Lira Participates in Endothelial Protection and Angiogenesis to Promote Diabetic Wound Healing

**Figure 5:**
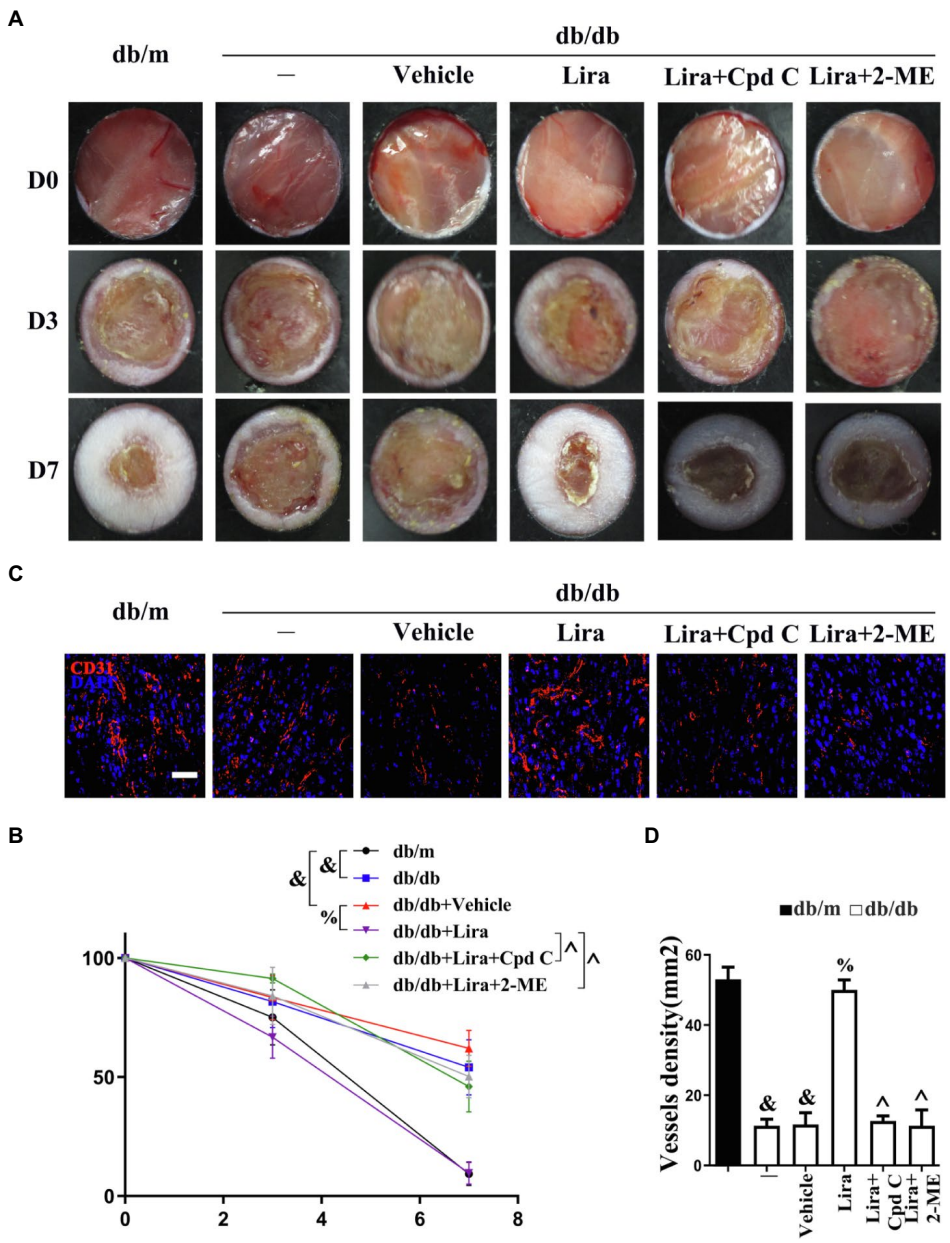
Liraglutide participates in endothelial protection and angiogenesis to promote diabetic wound healing. **(A)** Images of skin wounds and **(C)** confocal immunofluorescence with CD31 of wounded skin tissue sections, scale bars = 20 μm, from db/m mice, db/db mice, and db/db mice receiving Lira (100 nM) or vehicle treatment with saline smeared on the wound. For signaling pathway analysis, AMPK (10 μM) and 2-ME (5 μM) were injected intradermally into the wound edges in the mice, respectively, after Lira was smeared on the wound. **(B)** Quantification of the proportion of wound areas and **(D)** CD31-positive cells. All values displayed are means ± SEM of five independent experiments. ^&^*p* < 0.05 vs. db/m mice at D7; ^%^*p* < 0.05 vs. db/db mice or vehicle-treated db/db mice at D7; ^^^*p* < 0.05 vs. db/db mice receiving Lira at D7.

Considering Lira is a commonly used hypoglycemic agent, it is necessary to establish whether the effects of Lira involved a direct action on ECs, which is independent of its efficacy on blood glucose control. Endothelial cells are particularly important to angiogenesis, making key contributions to the wound healing process (Sawada et al., 2014; Chen et al., 2018). Thus, we examined the effect of topical Lira administration *in vivo* using a skin wound healing model in mice with type 2 diabetes mellitus (T2DM), a condition with a pathogenesis markedly involving endothelial cells. Relative to control db/m mice, CD31^+^ capillary density was confirmed to be reduced in the skin of db/db mice, whereas topical Lira treatment promoted CD31^+^ capillary density (Figures 5C,D). Meanwhile, the Lira treatment also accelerated wound healing in db/db mice (Figures 5A,B), without altering the fasting blood glucose level (Supplementary Figure S1). In addition, the role of Lira on non-diabetic wounds was also examined. We found that Lira could also accelerate wound healing as well as angiogenesis in the wound healing model of non-diabetic mice (Supplementary Figure S2).

**Figure 6:**
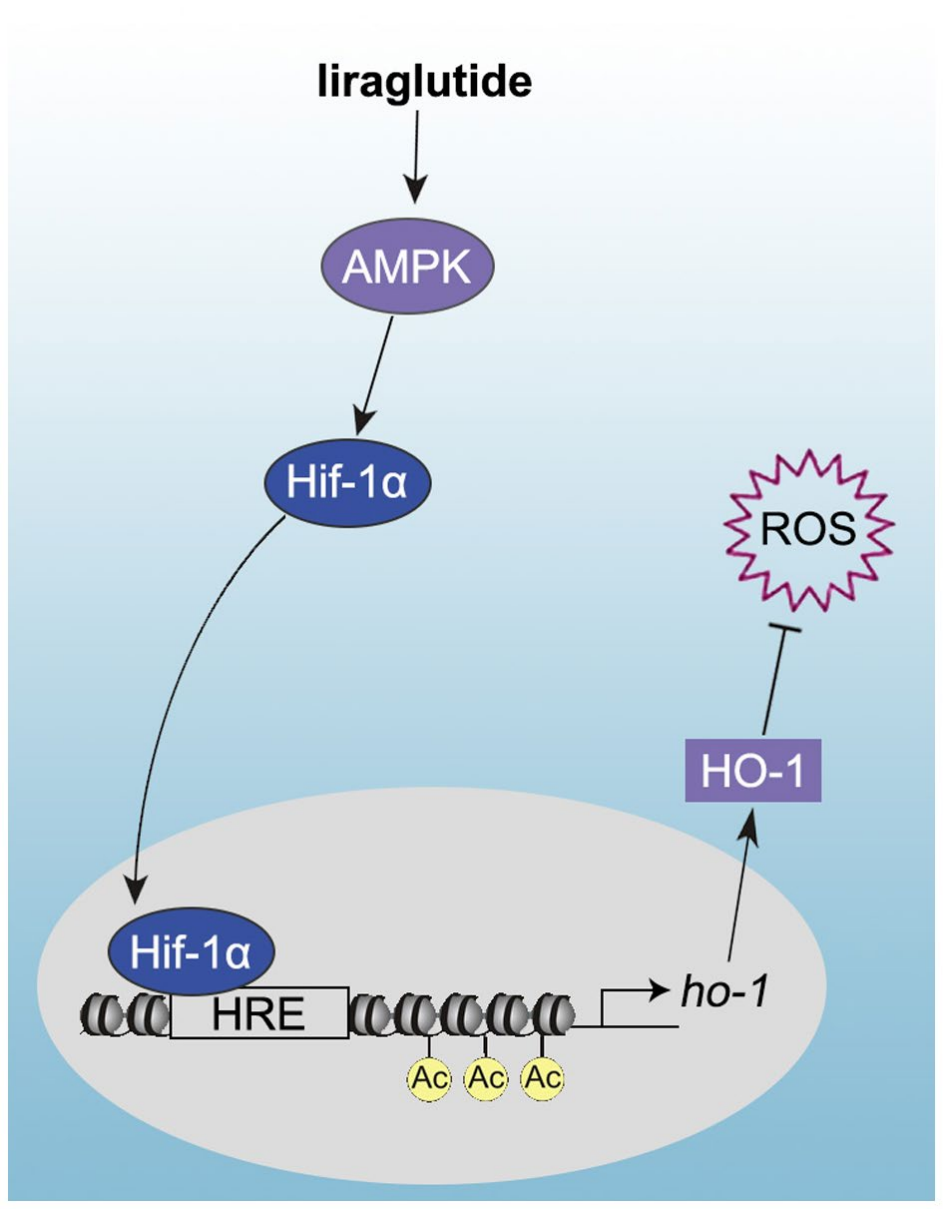
Schematic showing that the promotion of diabetic wound healing by Lira occurs *via* its AMPK-dependent endothelial protection and pro-angiogenic effects, which are regulated by the Hif-1α–HO-1 axis.

We then assessed the function of Lira-mediated AMPK-Hif-1α activation *in vivo*. The Lira-restored CD31^+^ capillary density in db/db mice was abrogated by Cpd C or 2-ME treatment (Figures 5C,D). Meanwhile, the Lira-accelerated wound healing in db/db mice was also abolished by Cpd C or 2-ME treatment (Figures 5A,B). The effects of inhibitors without Lira were also tested in the wound healing model. We found that Cpd C or 2-ME treatment alone in db/db mice had no obvious influence on the rates of wound healing from day 0 to 7 as compared with the vehicle-treated group. But from day 7 to 14, delayed wound healing was observed both in Cpd C and 2-ME-treated db/db mice, along with worse angiogenesis shown by significantly reduced CD31^+^ capillary density observed on day 14 (Supplementary Figure S3). These results demonstrated that the effects of Lira in promoting diabetic wound healing might be partly attributed to its pro-angiogenesis role *via* the AMPK-Hif-1α pathway.

## Discussion

This study provides novel evidence that Lira promotes diabetic wound healing in a mouse model of T2DM action against hyperglycemia impairment, at least in part, through inhibiting hyperglycemia-triggered endothelial dysfunction, which leads to angiogenic inhibition. This study provides evidence that Lira activates AMPK to participate in diabetic wound healing and that this process is regulated through the Hif-1α-HO-1 pathway. However, the present research has some limitations. The endothelial functions were mainly assayed in the aorta of diabetic mice and in HUVECs. Although the effect of the main interventions on these structures (aorta and HUVECs) were reproduced in the wound healing model, parameters that indicate endothelial function should be further addressed in capillary endothelial cells and injured skin tissues.

Vascular endothelial cells play a critical role in angiogenesis (Wils et al., 2017). The re-establishment of blood vessels through the process of angiogenesis is essential for the delivery of oxygen and nutrients to healing sites, making this process critical to wound healing (Zhao et al., 2017). Endothelial dysfunction is the earliest and most prominent change associated with the onset of diabetes and is also recognized as one of the initial events in the progression of many serious diabetic complications (Li et al., 2013). Several studies have highlighted the beneficial effects of Lira on diabetic endothelial dysfunction, establishing that Lira is a potential activator of AMPK (Balestrieri et al., 2015; López and Tena-Sempere, 2017). Many antidiabetic and vascular protective agents, including metformin, statins, and adiponectin, have been shown to prevent diabetic vascular dysfunction, possibly by activating AMPK in endothelial cells (Wassmann et al., 2006; Zou and Wu, 2008). The activation of AMPK in endothelial cells can trigger several biological effects that promote vascular homeostasis and angiogenesis, such as normalization of hyperglycemia-induced mitochondrial ROS production by induction of MnSOD and promotion of mitochondrial biogenesis (Kukidome et al., 2006), protection of hyperglycemia-induced endothelial cell apoptosis (Ido et al., 2002), improvement of the angiogenic functions of endothelial progenitor cells, and acceleration of diabetic wound healing (Yu et al., 2016). The results of this study confirm a broader role of AMPK as an important regulator of endothelial cells homeostasis and angiogenesis. To clarify the roles of AMPK in the endothelial protective action of Lira against HG conditions, we used AMPKα1/2 siRNA to interfere with AMPKα expression in HUVECs. Under AMPKα1/2 silencing, the pro-angiogenesis, anti-oxidative stress, and anti-apoptosis effects of Lira against HG impairment were abrogated. This result was further confirmed by the effect of the AMPK inhibitor Cpd C on HUVECs and the diabetes wound healing model derived from db/db mice.

AMP-activated protein kinase interacts with multiple substrates in addition to various metabolic enzymes, there are many transcription factors, such as Hif-1, p300, and hepatocyte nuclear factor 4 (HNF4), which mediate specific functions of AMPK (Leff, 2003). AMPK activity is critical for HIF-1 transcriptional activity and consequently expression of its target genes; an interaction between AMPK and Hif-1α was previously documented during pro-angiogenesis and in controlling cellular ROS levels (Abdel Malik et al., 2017; Rabinovitch et al., 2017). Hif-1α regulates the expression of genes involved in various cellular signaling pathways. Recent studies have found that the Hif-1α–HO-1 axis not only has an antioxidant function, but also has pro-angiogenesis activity (Choi et al., 2017; He et al., 2018). To activate HO-1 expression, HIF-1α must be transported to the nucleus. The present study demonstrated that HG-mediated AMPK activity inhibition increased cytoplasmic Hif-1α levels, whereas Lira-mediated AMPK activation facilitated Hif-1α translocation from the cytoplasm to the nucleus. In addition, the protein levels of Hif-1α and HO-1 were dramatically decreased under hyperglycemia, whereas they were increased by Lira.

Hypoxia, at least in part through activation of HIF-1α-related pathways, affects all steps of postischemic revascularization. Destabilization of HIF-1 has been reported as the most likely key event that transduces hyperglycemia into the loss of a cellular response to hypoxia in most diabetic complications (Bento and Pereira, 2011). To elucidate the roles of the Hif-1α–HO-1 axis in the endothelial protective effect of Lira against HG conditions, we used Hif-1α siRNA to interfere with Hif-1α expression in HUVECs. Under reduced Hif-1α expression, the pro-angiogenesis, anti-oxidative stress, and anti-apoptosis effects of Lira against HG impairment were indeed abrogated. This result was confirmed by treatment with the Hif-1α inhibitor 2-ME in HUVECs and the diabetes wound healing model derived from db/db mice.

## Conclusion

The present study elucidated the mechanism by which impairment of angiogenesis impedes healing of DFUs. The results of the current study have potential implications for both the pathogenesis and treatment of DFUs and other vascular complications associated with diabetes. Our data have revealed a novel pathway through which Lira prevents hyperglycemia-induced endothelial dysfunction and thus angiogenic impairment. This pathway, in which Lira activates AMPK, stimulates HO-1 expression through upregulation and promotion of the export of Hif-1α protein from the cytoplasm to the nucleus.

## Data Availability Statement

The original contributions presented in the study are included in the article/Supplementary material; further inquiries can be directed to the corresponding authors.

## Ethics Statement

The animal study was reviewed and approved by all animal experiments and methods performed in this study followed ethical guidelines for animal studies, and were approved by the Institutional Animal Care and Use Committee of Wenzhou Medical University after obtaining their ethical approval to pursue this study (wydw2020-0473).

## Author Contributions

HH, XH, and LW conceived the study, acquired data, interpreted the results, and drafted the manuscript. XH and HZ performed some cell experiments. HH, LW, MY, and FQ assisted technicians with animal sacrifice. CZ, MC, XW, and XC approved the final version. XW and XC designed the study, interpreted the results, and revised the manuscript. All authors contributed to the article and approved the submitted version.

## Conflict of Interest

The authors declare that the research was conducted in the absence of any commercial or financial relationships that could be construed as a potential conflict of interest.

## Publisher’s Note

All claims expressed in this article are solely those of the authors and do not necessarily represent those of their affiliated organizations, or those of the publisher, the editors and the reviewers. Any product that may be evaluated in this article, or claim that may be made by its manufacturer, is not guaranteed or endorsed by the publisher.
