# Supplementary material for: Liraglutide via Activation of AMP-Activated Protein Kinase-Hypoxia Inducible Factor-1α-Heme Oxygenase-1 Signaling Promotes Wound Healing by Preventing Endothelial Dysfunction in Diabetic Mice
Source: Front Physiol. 2021 Aug 16;12:660263. doi: 10.3389/fphys.2021.660263 (PMC8415222; doi:10.3389/fphys.2021.660263)
Supplement: Supplementary file 1 [file Data_Sheet_1.doc]

**Supplemental Material**

**Supplementary Figure 1**


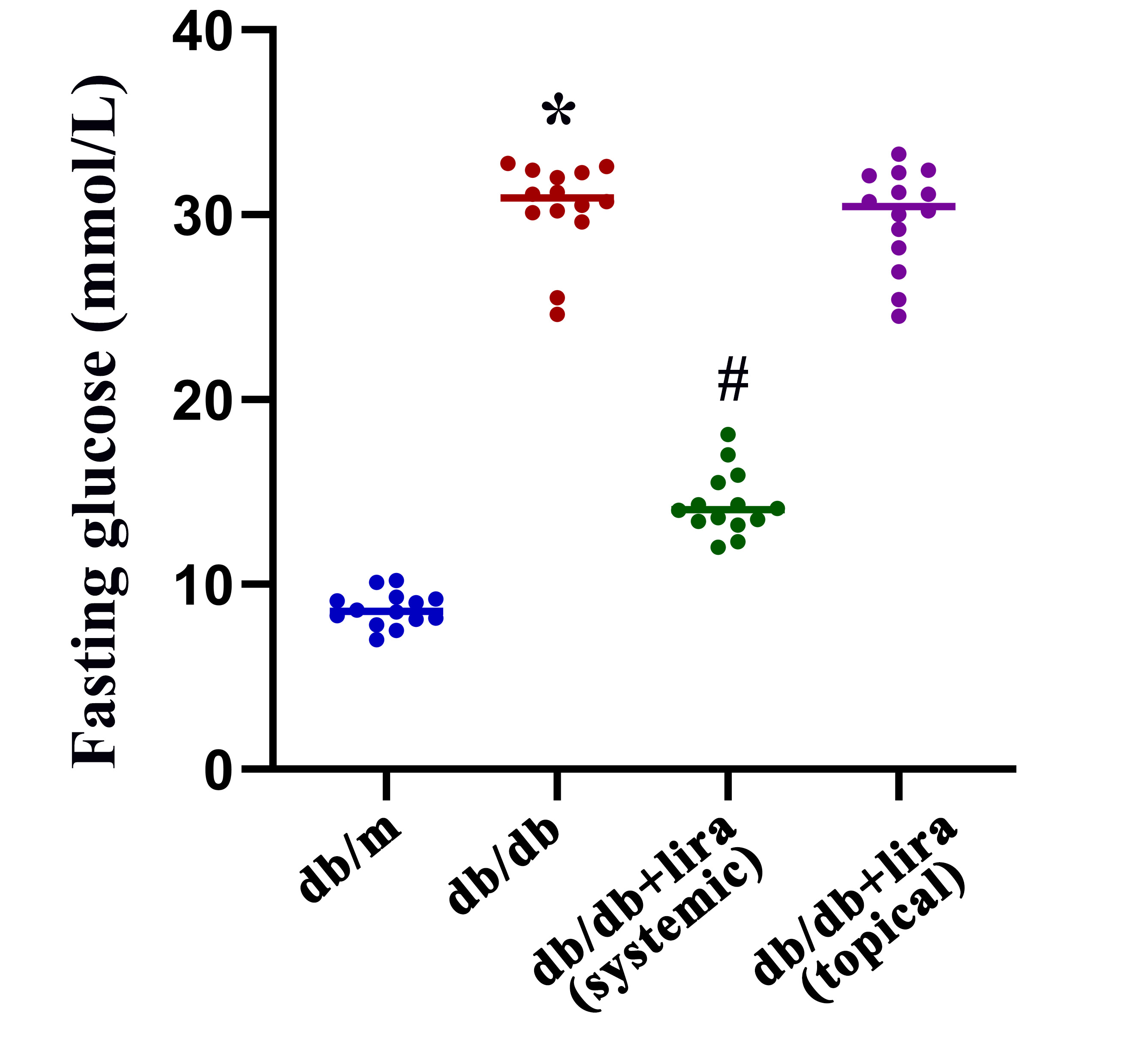


**Fig. S1:** The fasting blood glucose in db/m mice and diabetic db/db mice that were administered with long-term systemic (subcutaneously embedded osmotic minipumps) or topical (smeared on the wound) Lira administration. * p < 0.05 vs. db/m; # P < 0.05 vs. db/db (n = 14 in each group).

**Supplementary Figure 2**


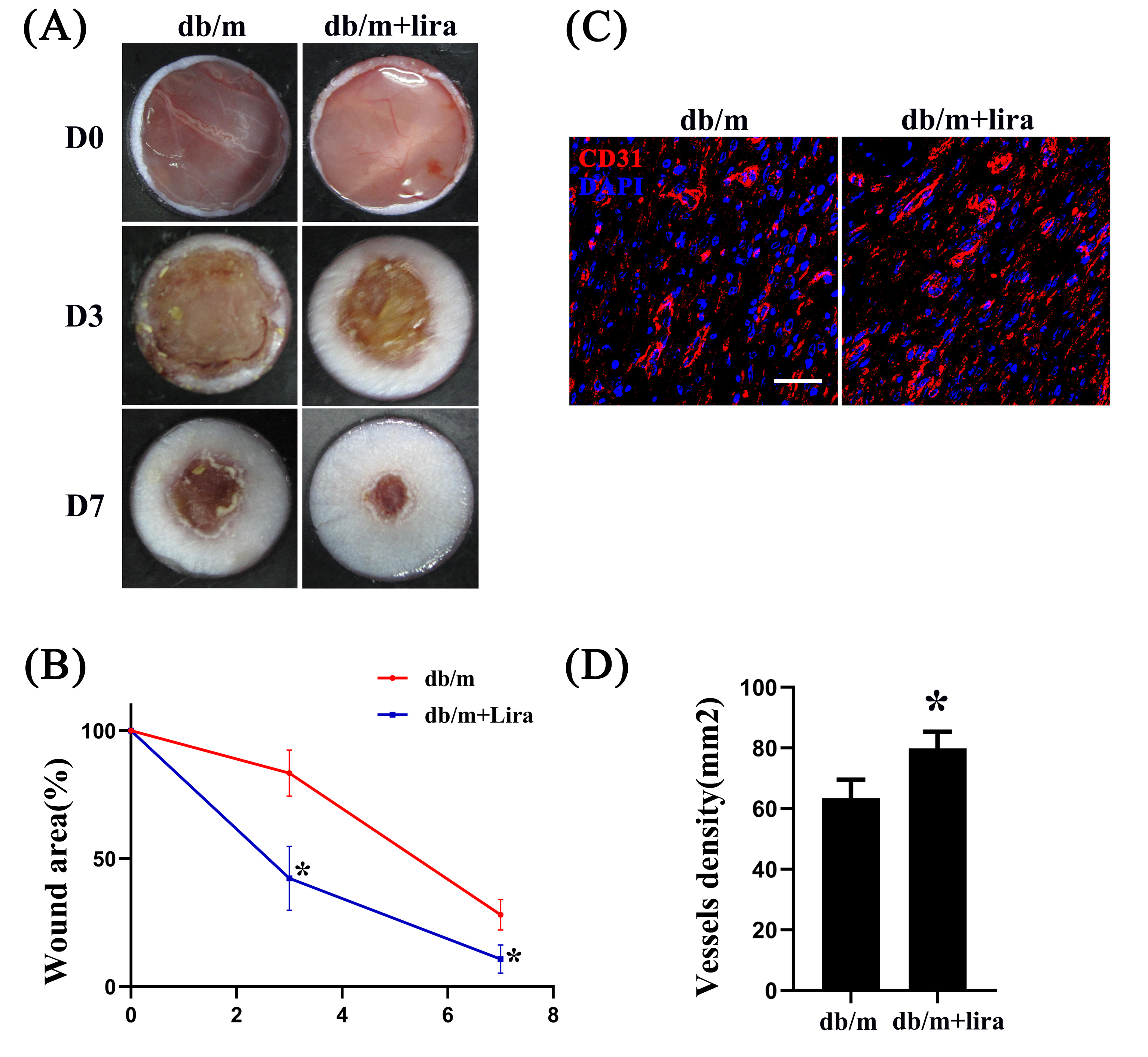


**Fig. S2: The effects of Lira on non-diabetic wounds.** (**A**) Images of skin wounds and (**C**) confocal immunofluorescence with CD31 of wounded skin tissue sections, scale bars=20 μm, from db/m mice, db/m mice receiving Lira (100 nM) smeared on the wound. (**B**) Quantification of the proportion of wound areas and (**D**) CD31-positive cells. All values displayed are means ± SEM of 5 independent experiments. * p < 0.05 vs. db/m mice.

**Supplementary Figure 3**


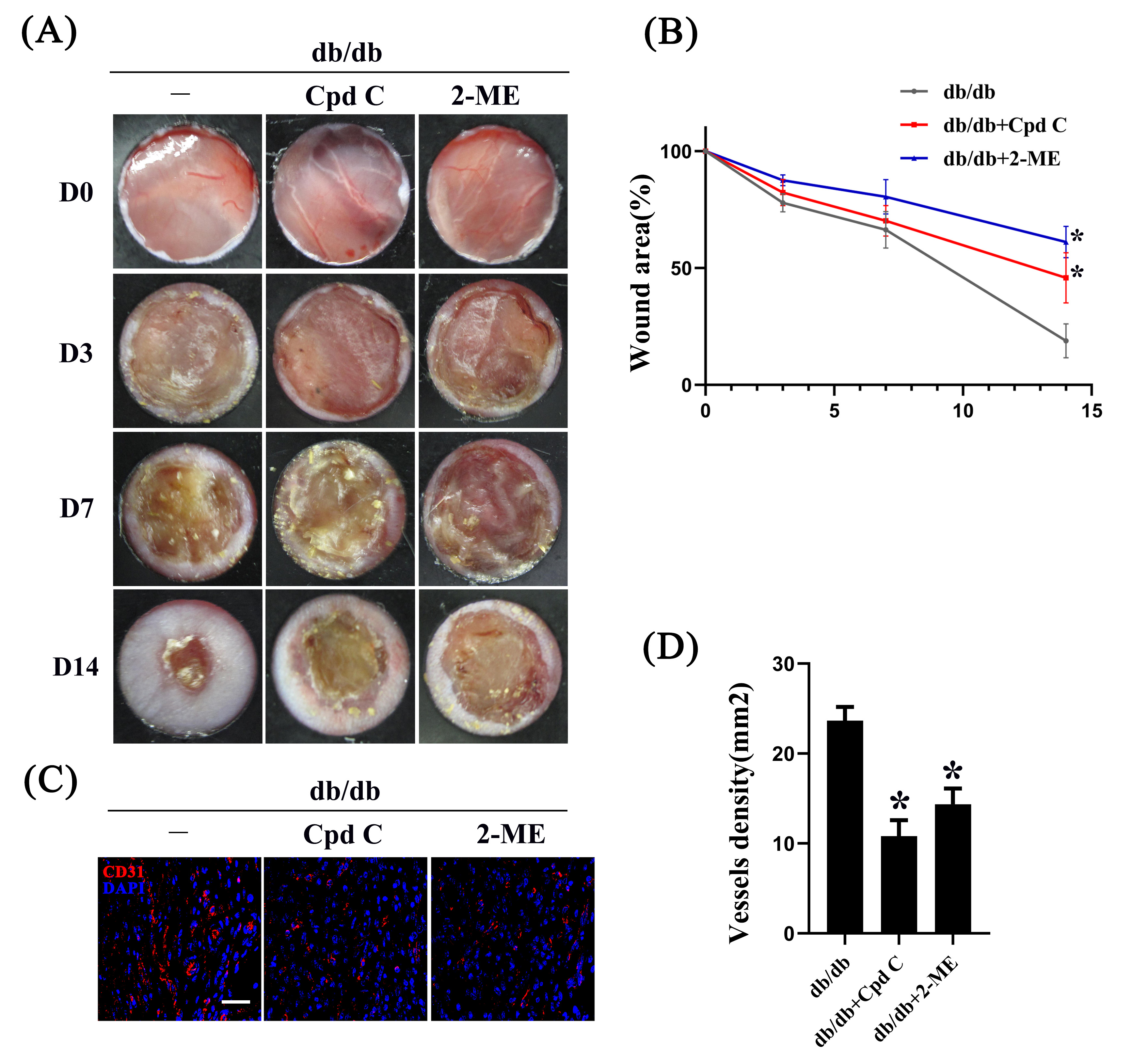


**Fig. S3: The effects of inhibitors without Lira on diabetic wounds.** (**A**) Images of skin wounds and (**C**) confocal immunofluorescence with CD31 of wounded skin tissue sections from db/db mice, scale bars=20 μm. For signaling pathway analysis, AMPK (10 μM) and 2-ME (5 μM) was injected intradermally into the wound edges immediately and 4 days after wounding. (**B**) Quantification of the proportion of wound areas and (**D**) CD31-positive cells. All values displayed are means ± SEM of 5 independent experiments. * p < 0.05 vs. db/db mice.

**Supplemental Methods**

**Immunoblot analysis**

Immunoblot analysis was conducted as described by our previous study ([Jia Sun](https://pubmed.ncbi.nlm.nih.gov/?size=20&term=Sun+J&cauthor_id=33360774) et al., 2021). Briefly, 30 μg protein from each sample was resolved by SDS–PAGE on Tris-Glycine gels, and transferred to polyvinylidene fluoride membrane. Membranes were blocked with 5% bovine serum albumin in Tris-buffered saline containing 0.1% Tween 20 (TBST) and incubated with primary antibodies overnight at 4°C. The following primary antibodies were used: cleaved-Caspase-3 (1:500; c-Caspase-3, Cell Signaling Technology, 9661), Bcl-2 (1:500; Abcam, ab59348), Bax (1:500; Abcam, ab32503), p-AMPKα (1:500; Cell Signaling Technology, 2535), AMPKα (1:500; Cell Signaling Technology, 2603), p-ACC (1:500; Cell Signaling Technology, 11818), 3-Nitrotyrosine (1:500; 3-NT, Abcam, ab61392), Hif-1α (1:500; Abcam, ab2185), and HO-1 (1:500; Cell Signaling Technology, 5853). Immunoreactive bands were visualized using Pierce ECL plus western blotting substrate (Thermo Scientific, 32132). ImageQuant 5.2 software (Molecular Dynamics, Chatsworth, CA) was used to quantify the expression of specific antigens, with the glyceraldehyde 3-phosphate dehydrogenase (GAPDH) (1:2000; Abcam, ab9485) and Lamin B1 (1:1000; Cell Signaling Technology, 12586) expression levels used as loading controls. n=5 for each experiment.

**In vitro angiogenesis (tube formation) assay**

Following the methods described by Sun et al ([Jia Sun](https://pubmed.ncbi.nlm.nih.gov/?size=20&term=Sun+J&cauthor_id=33360774) et al., 2021), in vitro HUVEC angiogenic activity was determined using a Matrigel tube formation assay, with capillary-like tubes images captured and analyzed with an EVOS computer-assisted microscope (Thermo Fisher Scientific Waltham, MA) and ImageJ software, respectively. n=5 for each experiment.

**Dihydroethidium staining assay of superoxide levels in HUVECs**

Briefly, fluorescent dihydroethidium (DHE) probes (Molecular Probe) (Sigma, D7008) were used to measure superoxide (O2−) generation according to the manufacturer’s instructions. n=5 for each experiment.

**Terminal deoxynucleotidyl transferase-mediated dUTP nick end labeling assay**

To assay apoptosis, HUVECs and aortic rings from each mouse (described later) were subjected to a terminal deoxynucleotidyl transferase-mediated dUTP nick end labeling (TUNEL) assay following the procedure outlined by Sun et al ([Jia Sun](https://pubmed.ncbi.nlm.nih.gov/?size=20&term=Sun+J&cauthor_id=33360774) et al., 2021) using the Cell Death Detection kit (Roche, 11684795910). n=5 for each experiment.

**Immunofluorescence staining of HUVECs and aortic ring sections**

HUVECs were grown on gelatinized coverslips overnight. Briefly, after the experimental procedure described above. The cells were fixed in 4% paraformaldehyde in PBS for 10 min and treated with 0.5% Triton for 15 min. The cells were then incubated with Hif-1α (1:500; Abcam, ab179483) at 4°C overnight. Then followed by Alexa fluor 647-conjugated anti-rabbit antibody (1:500; Abcam, ab150075) for 1 h at room temperature. Cellular nuclei were labeled with the fluorescent dye DAPI for 1 h. HUVECs cultured under each experimental condition were observed using the Leica TCS SP5 Confocal microscope (Leica, Wetzlar, Germany). n=5 for each experiment.

For aortic ring staining, 5-μm-thick sections were cut and incubated with anti-CD31 (1:500; Abcam, ab24590) and/or 3-NT (1:100; Abcam, ab61392) overnight at 4°C. After washing, samples were incubated with Alexa fluor 647-conjugated anti-mouse IgG secondary antibody (Abcam, ab150115) and/or Alexa fluor 488-conjugated anti-mouse IgG secondary antibody (Abcam, ab150113) at a dilution of 1:200 for 60 min at room temperature. Cell nuclei were labeled by DAPI. Digital images were acquired using the Leica TCS SP5 Confocal microscope. n=5 for each experiment.

**Isolation of nuclear and cytosolic extracts**

A Nuclear and Cytoplasmic Extraction kit (Millipore, 2900) was used to isolate nuclear and cytosolic extracts from HUVECs according to the manufacturer’s instructions.

**Transfection of siRNA into cells**

RNA interference was conducted according to the protocol from Santa Cruz Biotechnology (Dallas, TX). Cells were transfected with AMPKα1/2 siRNA (Santa Cruz Biotechnology, sc-45312), Hif-1α siRNA (Santa Cruz Biotechnology, sc-35561), or control scramble siRNA (Santa Cruz Biotechnology, sc-37007) using Lipofectamine 2000 reagent for 12 h in Opti-MEM medium. Prior to analysis in further experiments, transfected cells were transferred to full-growth medium for another 12 h. HUVECs were incubated with HG (33 mM) for 72 h with or without Lira (100 nM).

**Immunofluorescence staining of mouse wound healing**

Mouse wound tissue samples were sectioned, subjected to immunofluorescence staining with anti-CD31 (1:500; Abcam, ab24590) overnight at 4°C. After washing, samples were incubated with Alexa fluor 647-conjugated anti-mouse IgG secondary antibody (Abcam, ab150115) at a dilution of 1:200 for 60 min at room temperature, and observed using a Leica TCS SP5 Confocal microscope. n=5 for each experiment.

**Reference**

Sun, J., Huang, X., Niu, C., Wang, X., Li, W., Liu, M., et al. (2021). aFGF alleviates diabetic endothelial dysfunction by decreasing oxidative stress via Wnt/β-catenin-mediated upregulation of HXK2. *Redox. Biol*. 39, 101811. doi:10.1016/j.redox.2020.101811.
